# Exploring the Potential of Variational Autoencoders for Modeling Nonlinear Relationships in Psychological Data

**DOI:** 10.3390/bs14070527

**Published:** 2024-06-25

**Authors:** Nicola Milano, Monica Casella, Raffaella Esposito, Davide Marocco

**Affiliations:** Natural and Artificial Cognition Laboratory “Orazio Miglino”, Department of Humanistic Studies, University of Naples Federico II, 80133 Naples, Italy; nicola.milano@unina.it (N.M.); raffaella.esposito3@unina.it (R.E.); davide.marocco@unina.it (D.M.)

**Keywords:** machine learning, variational autoencoders, factor analysis, dimensionality reduction

## Abstract

Latent variables analysis is an important part of psychometric research. In this context, factor analysis and other related techniques have been widely applied for the investigation of the internal structure of psychometric tests. However, these methods perform a linear dimensionality reduction under a series of assumptions that could not always be verified in psychological data. Predictive techniques, such as artificial neural networks, could complement and improve the exploration of latent space, overcoming the limits of traditional methods. In this study, we explore the latent space generated by a particular artificial neural network: the variational autoencoder. This autoencoder could perform a nonlinear dimensionality reduction and encourage the latent features to follow a predefined distribution (usually a normal distribution) by learning the most important relationships hidden in data. In this study, we investigate the capacity of autoencoders to model item–factor relationships in simulated data, which encompasses linear and nonlinear associations. We also extend our investigation to a real dataset. Results on simulated data show that the variational autoencoder performs similarly to factor analysis when the relationships among observed and latent variables are linear, and it is able to reproduce the factor scores. Moreover, results on nonlinear data show that, differently than factor analysis, it can also learn to reproduce nonlinear relationships among observed variables and factors. The factor score estimates are also more accurate with respect to factor analysis. The real case results confirm the potential of the autoencoder in reducing dimensionality with mild assumptions on input data and in recognizing the function that links observed and latent variables.

## 1. Introduction

In psychometric research, the objects of study, such as personality traits, motivation, or attitudes, are often not directly observable or measurable. Consequently, the structure and effects of these unobservable variables, referred to as “latent variables”, are typically investigated through specific techniques like factor analysis. These methods aim to statistically relate the covariation between observed variables to latent variables [1].

Factor analysis, originally developed by Spearman [2], is a popular and widely used multivariate technique. Its goal is to approximate the original observed variables of a data set by linear combinations of a smaller number of latent variables called factors. In psychological research, factors are fundamental as they reduce dimensionality and represent psychological constructs strongly tied to psychological theory [3]. Indeed, factor analysis is mostly used to assess the degree to which the items on a scale conform to a theoretically indicated higher-order structure, investigating the construct validity and internal validity of psychometric tests [4].

However, factor analysis relies on a set of assumptions that may not always hold in psychological data [5]. Notably, it assumes that the relationships between indicators and factors are linear. The use of factor analysis for theory validation implies that the theory behind the factor-indicator relationship must assume linearity. Hence, it constrains the kinds of theories that can be validated through factor analysis.

In cases where nonlinear relationships exist, this assumption can lead to biased results and misleading interpretations [6]. Bauer [6] and Belzak and Bauer [7] highlighted two significant consequences of excluding nonlinear relationships. Firstly, it can result in the rejection of measurement invariance tests, and secondly, it can lead to the misidentification of nonlinear effects as interaction effects. Bauer [6] demonstrated that even a slight curvature in the relationship between a single indicator and a factor could cause metric and scalar invariance tests to be rejected. The rejection occurs because the data from different groups cover different regions of the nonlinear function, resulting in different slopes and intercepts when fitting a linear factorial model, leading to the rejection of invariance tests. Additionally, failure to account for nonlinear effects can confound the identification of interaction effects, as shown by Busemeyer and Jones [8] and later by Belzak and Bauer [7].

It is important to consider that items with nonlinear relationships may deviate from the assumptions underlying traditional psychometric methods. As a result, these items are often excluded during the initial stage of test construction or validation, even though they may contain valuable information about the latent variable being measured. Therefore, including them in the analysis could enhance the overall performance of the hypothesized model and provide a deeper understanding of the construct.

In this context, the availability of analysis techniques that do not rely on the assumption of linearity and allow for the exploration of the relationship between items and factors while accommodating nonlinearities could significantly improve psychometric research. Such techniques would enable researchers to fully explore the nature of the relationships within the data and capture the complex dynamics that may exist.

Various nonlinear factor analysis techniques have been proposed over the years. When considering nonlinearity in latent variable models, it is crucial to examine how and where the nonlinearity is modeled. Specifically, nonlinearity can exist in the relationship between items and factors or in the relationship between factors, referring to the measurement model or the latent model, respectively. The focus of this study is on the former kind of relationship.

To the best of our knowledge, although some studies have proposed nonlinear factor analysis models, the focus has primarily been on nonlinear relationships among factors, with a few exceptions. Since McDonald’s [9] pioneering work, new estimation methods for nonlinear factor analysis models have been developed successively. Some of these include the maximum-likelihood-based methods proposed by Klein and Moosbrugger [10], Klein and Muthén [11], Lee and Zhu [12], and Yalcin and Amemiya [13]. Other relevant methods include the method of moments introduced by Wall and Amemiya [14] and the Bayesian approaches developed by Arminger and Muthén [15] and Zhu and Lee [16]. However, the proposed methods to estimate nonlinear factor models are computationally demanding and require specialized techniques. Unlike linear factor models that often have closed-form solutions, nonlinear models typically involve iterative optimization algorithms. These algorithms aim to estimate the model parameters by minimizing the discrepancy between the observed data and the model’s predicted values. The complexity of nonlinear optimization routines, combined with the potential presence of local optima, can pose challenges in achieving convergence and obtaining reliable estimates.

Furthermore, these techniques are rarely used because they are complex to implement and interpret. Nonlinear relationships are often more nuanced and complex than their linear counterparts, making the identification and meaningful interpretation of latent factors more intricate. Researchers must carefully interpret the nature and direction of nonlinear associations, considering the specific functional form employed in the analysis. This interpretation process requires expertise and a deep understanding of the underlying constructs.

Despite these complexities, nonlinear methods hold great potential for advancing psychometric research, as they provide a means to capture the intricacies and nonlinear dynamics present in psychological constructs.

One interesting solution to address the issue of nonlinearity lies in the use of artificial neural networks (ANNs). ANNs are computational models inspired by the structure and function of biological neurons. They possess the ability to learn relationships between input and output variables automatically through training data without the need for manually programmed decision rules. This characteristic enables ANNs to extract relevant features from raw input data, eliminating the requirement for manual feature engineering [17]. Consequently, ANNs can handle complex and nonlinear patterns within the input-output relationship.

Unlike traditional psychometric methods that rely on predefined notions about the data’s nature and variables’ relationships to create data models, ANNs do not assume a specific function or relationship between variables. Instead, they operate as algorithmic models aiming to find the function that maximizes predictive power for a given dataset.

Autoencoders, a type of ANN, have been extensively studied for their capacity to reduce the dimensionality of input data [18,19]. Their aim is to reconstruct the input data and encode the most relevant information for input reconstruction in a smaller central layer, known as the “bottleneck layer” [20]. While perfect reconstruction of the input vectors is not possible due to the smaller size of the bottleneck layer, the central neurons of the autoencoder are associated with the intrinsic dimensionality of the data [21]. In certain conditions, autoencoders can converge to the solution of principal component analysis (PCA) [22].

Among the variants of autoencoders, the variational autoencoder (VAE) is of particular interest and has garnered attention in psychometric research [23,24]. VAE is a generative model that aims to describe how a dataset is generated in terms of a probabilistic model. It differs from traditional autoencoders as it imposes restrictions on the distribution of the central nodes, encouraging the latent space of the VAE to be independent and follow a predefined distribution, often a normal distribution [25]. This characteristic makes the latent space of VAEs more interpretable than that of simple autoencoders. The structured latent space of VAEs can also be used for data generation.

In this study, our primary objective is to employ variational autoencoders as a tool for identifying nonlinear relationships between items and the underlying factors. Our research goal is to identify the relationship between the items and the underlying factors without having an a priori idea about this relationship. We remain within the framework of classical test theory and compare factor analysis and variational autoencoders using different datasets, real and simulated.

In the initial phase of our work, we focus on analyzing the characteristics of autoencoder solutions using two synthetic datasets. The first synthetic dataset demonstrates a linear relationship between items and a single factor. Here, our aim is to evaluate whether the variational autoencoder’s solution converges toward a similar outcome as that of linear factor analysis. The second synthetic dataset illustrates a nonlinear relationship between a varying number of items and the factor. Specifically, the relationship between items and the factor follows a sigmoidal function. In this scenario, our hypothesis is that the autoencoder will yield more accurate estimates of factor scores and effectively capture the shape of the relationships between the input variables and the factor. At this point, we also investigate the generalizability of our results using a simulated dataset based on a two-factor model.

Furthermore, as we will provide an illustrative example of applying VAEs to a real-world dataset, demonstrating their applicability and relevance in practical scenarios.

Thus, we propose autoencoders as a valuable tool for exploring the relationship between observed and latent variables, enabling dimensionality reduction while accounting for nonlinear relationships.

This work is structured as follows. First, we discuss the use of artificial neural networks and autoencoders in psychometrics research, offering an overview of the existing literature. Next, we present the technical details of variational autoencoders and describe how the data used in this study were simulated. We then proceed to present our analyses and discuss the results derived from the application of the VAE model to the synthetic datasets. At this point, we show an illustrative example of an application on a real dataset. Finally, we offer concluding remarks that summarize the key findings and discuss the implications of employing VAEs in psychometric research.

## 2. Artificial Neural Networks in Psychometrics

Artificial neural networks have proven to be an advantageous predictive methodology in psychometrics, both in applied contexts and in the realm of methodology research. ANNs can easily master very large and different types of data, removing some constraints that characterize more traditional techniques of data analysis, especially when applied to behavioral recorded data, which are often noisy, large in quantity, and structured in a complex temporal fashion.

In the realm of psycho-diagnosis, for example, Linstead et al. [26,27] used neural networks to predict the extent to which children with autism spectrum disorders would benefit from early behavioral interventions. A recent study by Perochon et al. [28] automated the detection of ASD by analyzing motion features detected during a task performed on a tablet, assuming that motor abnormalities may be a potential hallmark of ASD [29]. Milano et al. [30], using a variational autoencoder, showed that the motion features of children with autism differ consistently from those of children with typical development. ANNs have also been used to enhance the diagnosis of psychological disorders, as shown in a review by Kaur and Sharma [31]. Growing evidence suggests that artificial intelligence approaches to classify psychiatric patients offer superior predictions of treatment outcomes compared with traditional DSM/ICD-based diagnoses [32].

From a methodological perspective, as noted by Lanovaz and Bailey [33], artificial neural networks have been used for the development and evaluation of psychological theories [34,35], for behavior measurement [36,37,38], and within the Item Response Theory framework [39,40]. Furthermore, many tutorial works specifically targeted at psychologists about machine learning and neural networks have been proposed recently [17,41].

In particular, artificial neural networks have been used to select variables for inclusion in a psychopathological model [42] and for the development of short forms of tests [43]. Staying within the methodological realm, the demonstrated ability of autoencoders to extract essential information from data has paved the way for new applications of autoencoders. Urban and Bauer [23] introduced a novel deep learning-based variational inference (VI) algorithm using an importance-weighted autoencoder (IWAE) for exploratory item factor analysis (IFA). The IWAE can predict the log-likelihood of all possible responses on a Likert scale, enabling the retrieval of the five-factor structure of the Big Five model from a large Big5 dataset. In addition, Huang and Zhang [44] proposed a variational autoencoder (VAE) model to study the structure of personality tests, comparing it with linear factor analysis. In their work, they used autoencoders to derive the values of factor loadings. Finally, Esposito et al. [45] explored the use of autoencoders as a method to extract causal structure from psychometric data, showing that a nonlinear autoencoder has a greater ability than PCA to capture item–factor relationships.

## 3. Methods

### 3.1. Variational Autoencoder

In this section, we introduce the concept of variational inference, and then we explain how this statistical concept can be incorporated into a neural network, thus generating the variational autoencoder.

Latent variable models allow us to express observed data, x∈X, through unobserved, or latent, variables z∈Z. By doing this, we can better describe our data in terms of unobserved factors, assuming $\dim\left(Z\right)<\dim\left(X\right)$.

In the variational inference framework, we suppose that ***x*** is an observation from our dataset and assume that ***x*** is generated from the unobserved latent variable ***z***. The generative process of ***x*** can be summarized in two steps: First, a latent variable ***z*** is sampled from the prior distribution *p*(*z*); second, the data ***x*** is sampled from the conditional likelihood distribution *p*(*x|z*). At this point, we can use the Bayes’ theorem to connect the prior *p*(*z*) and the likelihood distribution *p*(*x|z*):

(1) $$p\left(z|x\right)=\frac{p\left(x|z\right)p\left(z\right)}{p\left(x\right)}$$

where *p*(*z|x*) is the posterior probability that we are looking for, and *p*(*x*) is the marginal probability. In order to sample from *p*(*z|x*), we need to compute the marginal *p*(*x*) integrating out the unobserved variables:

(2) $$p\left(x\right)=\int_{z}dz\;p\left(\left.x\right|z\right)p\left(z\right)$$

This integral has a time complexity exponential in dim(Z) and is often intractable even for simple conditionals like a mixture of Gaussians. To circumvent the direct computation of *p*(*x*) in variational inference (VI), the model posterior is approximated by a family of simpler distributions, *Q*, that allows efficient evaluation and sampling and are as close as possible to the true posterior. The problem is to find an approximating distribution $q_{\theta }\left(z\right)\in Q$, where θ are the variational parameters that are as close as possible to the true latent variable posterior. The canonical choice is the reverse KL divergence between ${KL(q}_{\theta }\left(z\right)||p\left(z|x\right))$:

(3) $${\theta^{*}}_{x}={argmin}_{\theta }E_{q_{\theta }\left(z\right)}\left[log\frac{q_{\theta }\left(z\right)}{p\left(z|x\right)}\right]$$

The final optimized version of $q_{\theta^{*}}\left(z\right)$ can be used instead of the true posterior. Because we do not know the true posterior, we cannot directly optimize ${KL(q}_{\theta }\left(z\right)||p\left(z|x\right))$, so we have to use Bayes’ theorem to define the problem in terms of evaluable quantities:

(4) $${KL(q}_{\theta }\left(z\right)||p\left(z|x\right))=E_{q_{\theta }\left(z\right)}[\;\log\;q_{\theta }\left(z\right)-\log\;p(z|x)]\;=E_{q_{\theta }\left(z\right)}[\;\log\;q_{\theta }\left(z\right)-(\log p\left(x,z\right)-\log p\left(x\right))]\;=\log p\left(x\right)-F\left(x;\theta \right)$$

where the variational free energy *F* is defined as follows.

(5) $$F\left(x;\theta \right)=\int dz{\;q}_{\theta }\left(z\right)\left(\log p\left(x,z\right)-\log q_{\theta }\left(z\right)\right)\;\;$$

The free energy sets a lower bound on $log\;p\left(x\right)$ due to the non-negativity of the KL-divergence, so it is common to refer to $F\left(x;\theta \right)$ as the evidence lower bound (ELBO), and the likelihood *p*(*x*) is also referred to as the evidence. Because the evidence is a constant with respect to the variational parameters θ, maximizing the ELBO is equivalent to minimizing the KL-divergence ${KL(q}_{\theta }\left(z\right)||p\left(z|x\right))$.

Variational autoencoder (VAE) [25] is a latent variable model that applies variational inference, i.e., maximizes the ELBO throughout artificial neural networks, making the problem differentiable for the backpropagation training. It is composed of two symmetrical neural networks, an encoder and a decoder, connected by a hidden layer that maps the inputs in a low-dimensional space, usually referred to as latent space. Differently from traditional autoencoders, which directly encode the inputs *x* into latent points z in a deterministic way and then decode these points to reconstruct *x*, VAE is formed by a probabilistic encoder, defined by p(z|x), that describes the distribution of the encoded variable given the input *x*. This process enables us to define a conditional model p(x|z) that describes how observed data depends on latent variables, as well as a prior density *p*(*z*) over the latent space.

**Figure 1:**
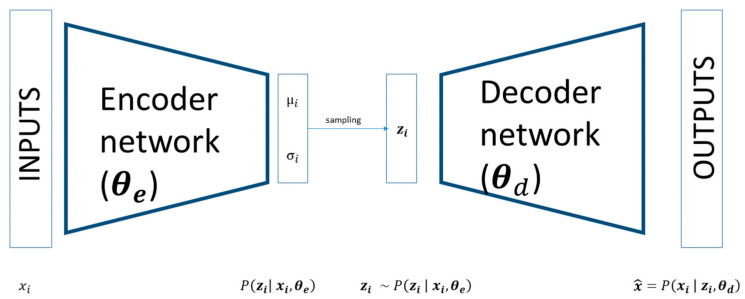
Graphical representation of the variational autoencoder.

The encoder part of a VAE models the distribution $q\left(z_{i}\right)=P\left(z_{i}\;\right|\;x_{i},\theta_{e})$, where $x_{i}\in \;R^{n}$ are the inputs, the parameters of the distribution $\theta_{e}$ are the weights of encoder layers, and $z_{i}\in R^{p}$ are the latent variables. As shown in Figure 1, the outputs of the encoder are the parameters of the conditional distribution $P\left(z_{i}\;\right|\;x_{i},\theta_{e})$. This conditional distribution is usually defined by *m* numbers of parameters. Consequentially, we will have *m* set of output neurons from the encoder. The dimension of this set depends on the parameters. For example, if the latent space is *p*-dimensional, and the distribution is a multivariate Gaussian distribution, we have two sets of output neurons, the *p* neuron referring to the mean ${(\mu }_{z|x})$ and (*p x p*) neuron for the covariance matrix ($\Sigma_{z|x}$) of this distribution. For diagonal distribution, the number of neurons for the covariance is reduced to *p*. In principle, any distribution with any number of parameters can be chosen to model $P\left(z_{i}\;\right|\;x_{i},\theta_{e})$, but a typical choice is the multivariate Gaussian with diagonal covariance:

(6) $$\;\;q\left(z_{i}\right)=P\left(z_{i}\;\right|\;x_{i},\theta_{e})=N\left(z_{i}|\mu_{z|x},\Sigma_{z|x}\right)\;$$

So, when a sample $x_{i}$ is given as input to the encoder, the parameters of the conditional distribution $q\left(z_{i}\right)$ are obtained. We can sample the corresponding latent variables from the distribution of the latent space:

(7) $$z_{i}\;∼q\left(z_{i}\right)=P\left(z_{i}\;\right|\;x_{i},\theta_{e})\;\;$$

**Figure 2:**
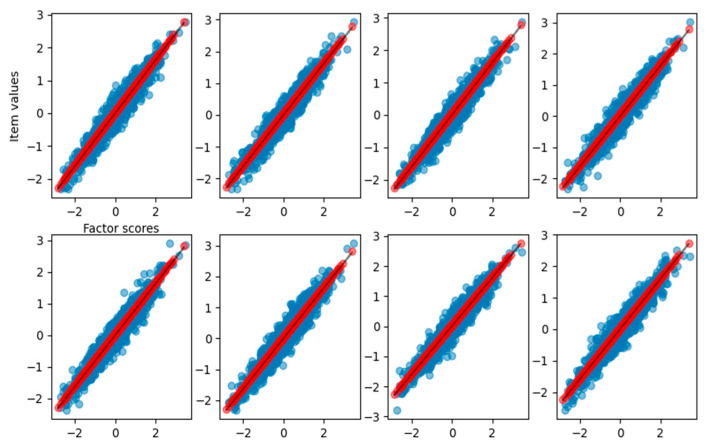
Graphical representation of the relationship between the factor scores and the eight-item values for the FA reconstruction of the linear case dataset. Blue dots are the true item values, red points are the reconstruction from factor analysis, and the black solid line is the result of the curve fitting.

As shown in Figure 2 this latent variable is then passed to the decoder part of the network that tries to reconstruct the input starting from a compact latent representation. Sampling from the latent space is reduced to one single MonteCarlo draw in the base version of VAE, and other methods have been proposed to reduce the noise, augmenting the number of samplings from the latent space [46]. The structure of the decoder is symmetrical with respect to the encoder and models a conditional distribution $P\left(\tilde{x_{i}}\;\right|\;z_{i},\theta_{d})$, where $\theta_{d}$ are the weights of the decoder layers. The input and output of the decoder are $z_{i}\in R^{p}$ and $\tilde{x_{i}}\in \;R^{n}.$ The outputs of the decoder can directly compute the reconstructed data or determine the parameters of the conditional distribution $P\left(\tilde{x_{i}}\;\right|\;z_{i},\theta_{d})$, just like in the encoder case; usually, the former is more common, so the decoder tries to reconstruct the input as well as possible.

In VAE, to overcome the problem of computing the derivative through $P\left(z_{i}\right|x_{i},\theta_{e})$, when sampling from the latent space, the reparameterization technique is used [25]. Sampling from latent space blocks the gradient flow, so we assume that $z_{i}$ is a deterministic function of another random variable $\varepsilon_{i}$:

(8) $$z_{i}=g\left(\varepsilon_{i},x_{i},\theta_{e}\right)$$

where $\varepsilon_{i}$ is a random variable sampled from P(ε). If $\varepsilon_{i}$ and $z_{i}$ are considered univariate Gaussian variables, we have the following:

(9) $${\;\;z}_{i}\;∼N\left(µ,\sigma^{2}\right),\;\;\varepsilon_{i}∼N\left(0,1\right),\;\;{\;\;\;z}_{i}=µ+\sigma \varepsilon_{i}$$

Reparameterization relies on the ability to express the expectation over some distribution $q_{\theta_{e}}\left(z_{i}\right)$ parametrized by $\theta_{e}$ replacing $z_{i}$ with $g(\varepsilon_{i},x_{i},\theta_{e})$ and converting it into an expectation over the base distribution P(ε). Given this transformation, *g* became differentiable and invertible.

Finally, VAE is trained by backpropagation, where the backpropagation algorithm is used for training the weights of the encoder and decoder networks so that the whole network is trained together. If we denote the whole network weights with $\theta ={\{\theta }_{e},\theta_{d}\}$, backpropagation trains VAE using gradient descent to maximize the ELBO. The loss function is then composed of a reconstruction term and a regularization term that ensures the regularity of the latent space and the correct approximation to our chosen prior distribution. This regularization term is expressed as the Kullback–Leibler divergence between the returned latent distribution and, in our case, a diagonal Gaussian distribution prior with a 0 mean and standard deviation of 1.

(10) $$\;loss=L_{r}(x_{i},\tilde{x_{i}})+\;{KL(q}_{\theta }\left(z_{i}\right)|N\left(0,1\right))\;\;\;\;$$

where $L_{r}(x_{i},\tilde{x_{i}})$ is a reconstruction error of the data and ${KL(q}_{\theta }\left(z_{i}\right)|N\left(0,1\right))$ is the Kullback–Leibler divergence between our latent distribution and a diagonal Gaussian distribution with zero mean and unitary variance.

### 3.2. Data Simulation Process

The data used in this study consists of two simulated datasets generated using the approach described in Bauer [6]. In the following sections, we provide a more detailed description of each of them.

#### 3.2.1. Dataset with Linear Relationships

The first synthetic dataset is simulated according to the general factor model equation:

(11) $$x_{i}=\tau +\Lambda \xi_{i}+\varepsilon_{i}$$

where $x_{i}$ is a vector of scores for p manifest variables (or factor indicators) for individual i. The vector τ represents the indicator intercepts, and Λ is a matrix of factor loadings that describes the regression of the manifest variables on the q latent common factors in the q × 1 vector $\xi_{i}$. The vector $\varepsilon_{i}$ contains the residual variability of the indicators after accounting for the influence of the common factors. This residual variability includes both random measurement error and true-score variability that is specific to each indicator.

For this study, we simulated a single sample of 1000 subjects, considering a one-factor model. We generated a total of 8 observed variables. The specified population model has the following form:

(12) $$\left(\begin{array}{c} x_{1i}\\ x_{2i}\\ x_{3i}\\ x_{4i}\\ x_{5i}\\ x_{6i}\\ x_{7i}\\ x_{8i} \end{array}\right)=\left(\begin{array}{c} 0\\ 0\\ 0\\ 0\\ 0\\ 0\\ 0\\ 0 \end{array}\right)+\left(\begin{array}{c} 0.8\\ 0.8\\ 0.8\\ 0.8\\ 0.8\\ 0.8\\ 0.8\\ 0.8 \end{array}\right)\left(\xi_{i}\right)+\left(\begin{array}{c} \varepsilon_{1i}\\ \varepsilon_{2i}\\ \varepsilon_{3i}\\ \varepsilon_{4i}\\ \varepsilon_{5i}\\ \varepsilon_{6i}\\ \varepsilon_{7i}\\ \varepsilon_{8i} \end{array}\right)\;$$

$\xi_{i}$ was drawn from a normal distribution with a mean of 0 and a variance of 1. The residuals of the indicators were assumed to be normally distributed and independent, with a variance of 0.20 for each indicator. This assumption corresponds to setting the reliabilities of the linear indicators to 0.80. Indicators simulated with this method are continuous.

#### 3.2.2. Dataset with Nonlinear Relationships

In the second dataset used for this study, we have incorporated observed variables with nonlinear relationships to the latent factor. Specifically, the dataset consists of 8 observed variables. We have generated two alternative conditions. One in which only one item has a nonlinear relationship with the factor and the other in which four have linear relationships, as described earlier, and the remaining four have nonlinear relationships with the factor.

In general, for a single manifest variable $x_{i}$ and a latent factor ξ*_i_*, the measurement model is as follows:

(13) $$x_{i}=\tau +f\left(\Lambda {,\xi }_{i}\right)+\varepsilon_{i}$$

where $f(\Lambda {,\xi }_{i})$ is a nonlinear function, and ε denotes residuals. In this study, we define the nonlinear function as follows:

(14) $$f\left(\Lambda {,\xi }_{i}\right)=\frac{1}{\left(1+e^{{,-3*\xi }_{i}}\right)}\Lambda$$

$\xi_{i}$ was drawn from a normal distribution with a mean of 0 and a variance of 1. The residuals of the indicators were assumed to be normally distributed and independent, with a variance of 0.20 for each indicator. In this case, observed variables simulated with this method are continuous.

### 3.3. VAE’s Architecture and Performance Measures

In the specific VAE architecture used in this study, the encoder network consists of 3 fully connected layers. The input layer has 8 neurons (the number of observed variables), and the first hidden layer has 4 neurons and a RELU activation function. The central layer has 2 neurons, representing the mean and log-variance of the latent space. The decoder network also consists of three fully connected layers and is specular to the encoder.

The VAE also includes a reparameterization trick to allow the gradient to flow through the latent space. Instead of directly sampling from the learned latent distribution, the trick samples from a standard normal distribution scale the sample by the learned variance and shifts the sample by the learned mean. This results in a differentiable operation that can be used to train the model via backpropagation. The VAE loss function is a combination of the reconstruction error and the KL divergence regularization term. The reconstruction error is measured using the mean squared error (MSE) loss function. To train the autoencoder, we used Adam as an optimization algorithm with a learning rate of 0.001 for a total of 100 learning epochs. We used a K-fold cross-validation on the dataset with k = 10; we did not find a statistical difference in performance among the networks trained with different folds. For the analysis implemented in this work, we choose the network showing the best generalization performance. The network’s hyperparameters are obtained from a preliminary optimization process where we tested different numerosity of hidden neurons, different activation functions and learning rates.

To test the performance of the VAE and of the factor analysis in retrieving the factor scores, we used the Mean Average error (MAE) and Root Mean Squared Error (RMSE):

(15) $$MAE=\frac{\sum_{i=1}^{n}\left|\xi_{i}-\tilde{\xi_{i}}\right|}{n},\;\;RMSE=\sqrt{\;\frac{\sum_{i=1}^{n}{\left(\xi_{i}-\tilde{\xi_{i}}\right)}^{2}}{n}}$$

where $\xi_{i}$ and ($\tilde{\xi_{i}}$) are the true and the estimated factor scores, respectively, and n is the sample size. To test the ability in reconstructing the true item values we reported the explained variance of the two methods.

## 4. Results

The Results section is structured as follows: First, we test the VAE and factor analysis with the linear dataset, where the items have a linear relationship with their underlying factor. Next, we evaluate both methods using datasets with nonlinear relationships. The simulated data results conclude with a paragraph that compiles all the previous results and provides an extension using a two-factor dataset. Finally, we test the FA and VAE in a real-world scenario, as discussed in the final paragraph of the Results section.

### 4.1. Linear Case

For the first series of experiments, we use the dataset described by Equation (11), and then we perform a factor analysis to estimate the loadings and the factor scores ($\tilde{\xi_{i}}$) and reconstruct the item values ($\tilde{x_{i}}$). Formally, we can connect these two quantities:

(16) $$\tilde{x_{i}}=f\left(\tilde{\xi_{i}}\right)$$

The *f* function ideally should be equivalent to Equation (11), where the slope of *f* is the loadings Λ of each item. To verify this hypothesis, we fit *f* with the linear Equation (11) using the least squares method, leaving as a free parameter the loadings Λ. Table 1 reports the values and the standard deviation obtained from the fitting and the loadings and communalities directly derived from the FA:

As shown in Table 1, the slope of the curve obtained from the fitting process matches the loadings used to generate the dataset. In this case, we achieve a total explained variance of 0.92. To visualize the relationship between the reconstructed item values and the estimated factor scores, Figure 2 reports the observed item response with respect to the true factor scores (blue dots), the reconstructed items with respect to estimated scores (red dots), and the result of curve fitting (black solid line).

**Figure 3:**
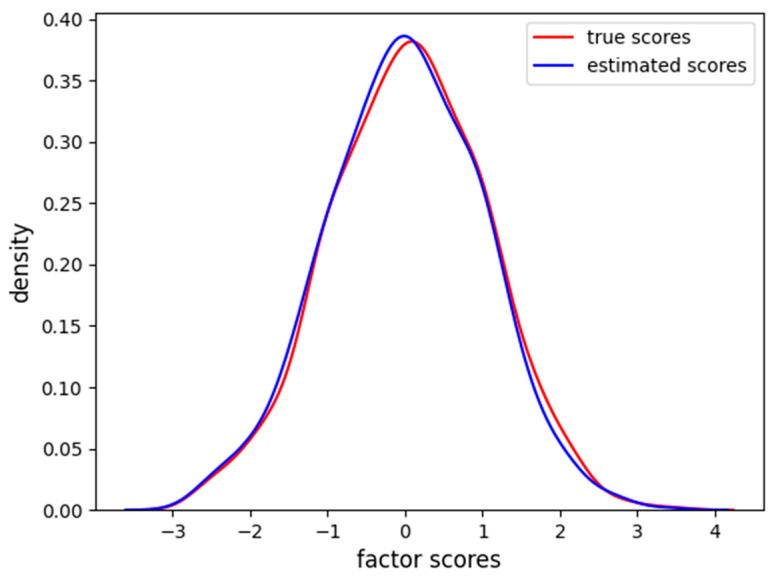
Density of true and estimated factor scores for the FA scores in the linear case scenario. The curve follows a Gaussian distribution with mean zero and unitary variance.

In Figure 2, it is clear how FA captures the linear relationship between the factor scores and each item. In Figure 3, we also report the distribution of the factor scores for the true and estimated scores. Figure 3 shows how the density of estimated scores from FA correctly resembles the true Gaussian distribution of the factor scores.

We measured an MAE of 0.11 and 0.15 RMSE between the two distributions. For the variational autoencoder, we repeated the same analysis. We connected the reconstruction $\tilde{x_{i}}$ and the estimated scores $\tilde{\xi_{i}}$ through the linear function *f* and performed a least-squares fitting to find the slope of the curve representing the factor loadings. Results are reported in Table 2.

**Figure 4:**
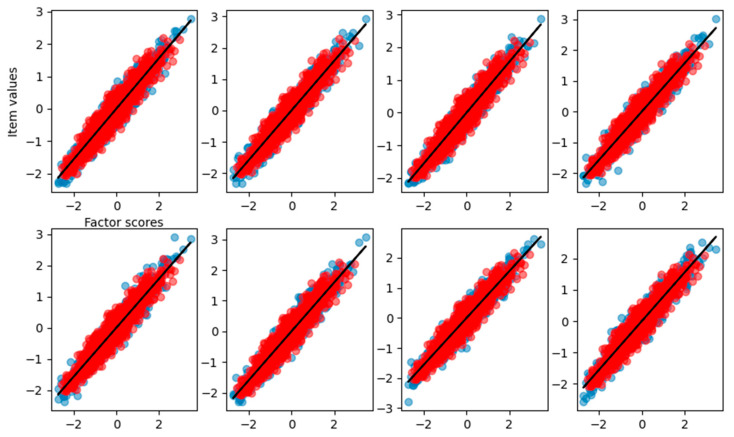
Graphical representation of the relationship between the factor scores and the eight-item values for the VAE reconstruction of the linear dataset. Blue dots are the true item values, red points are the reconstruction from factor analysis, and the black solid line is the result of the curve fitting.

The VAE, just like the FA, correctly reconstructs the relationship between factor scores and item values with an explained variance of 0.91. In Figure 4, the graphical reconstruction is reported.

**Figure 5:**
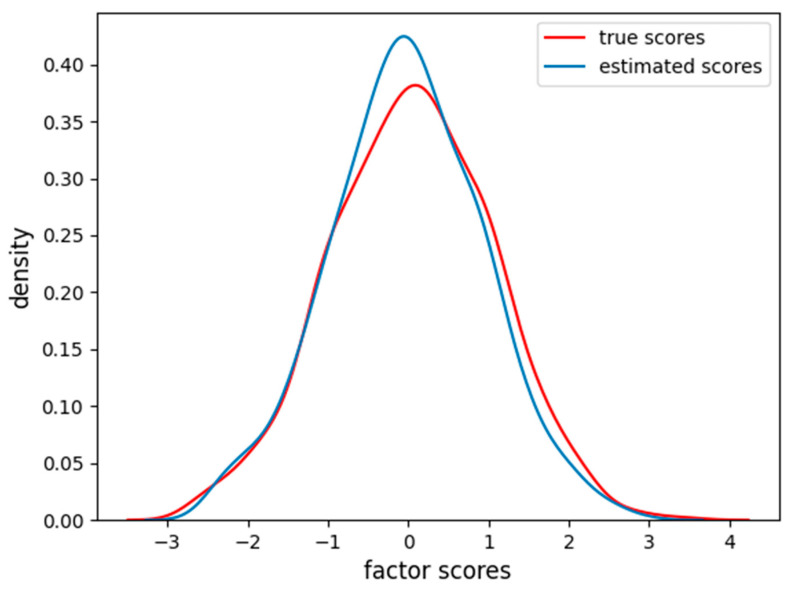
Density of true and estimated factor scores for the VAE in the linear case scenario. The curve follows a Gaussian distribution with mean of zero and unitary variance.

From Figure 4, we can see that the VAE, differently from the FA, also reconstructs the noise in the relationship between factor scores and item values. This is due to the fact that sampling from the latent space with a single MonteCarlo draw is subject to Gaussian noise, and the reconstruction results are more scattered with respect to the FA. Nevertheless, the noise does not affect the correct reconstruction of the function used to specify the relationship between the factor and the items. The density of factor scores estimated from the VAE, along with the true scores density, is also reported in Figure 5.

The VAE correctly finds the Gaussian distribution of the factor scores as imposed by the regularization on the VAE latent space. The MAE between the true and estimated scores is 0.13, and the RMSE is 0.17, a little bit higher than FA in the linear setting.

These findings show that a VAE with a single hidden layer correctly finds the underlying relationship between factor scores and observed items in a linear scenario, retrieving the same loadings of the FA.

### 4.2. Nonlinear Case

In this section, we modify the dataset by injecting one or more items with a nonlinear relationship to their underlying factor, as described by Equation (13). The fitting process is the same as described above, with the difference that we use for the function *f*, representing the connection between items and scores in the nonlinear equation.

In the first experiment that we conducted, item number eight of the dataset was modified to have a nonlinear relationship with the factor. The loadings and communalities estimated from the FA are reported in Table 3, along with the parameters estimated from the fitting.

**Figure 6:**
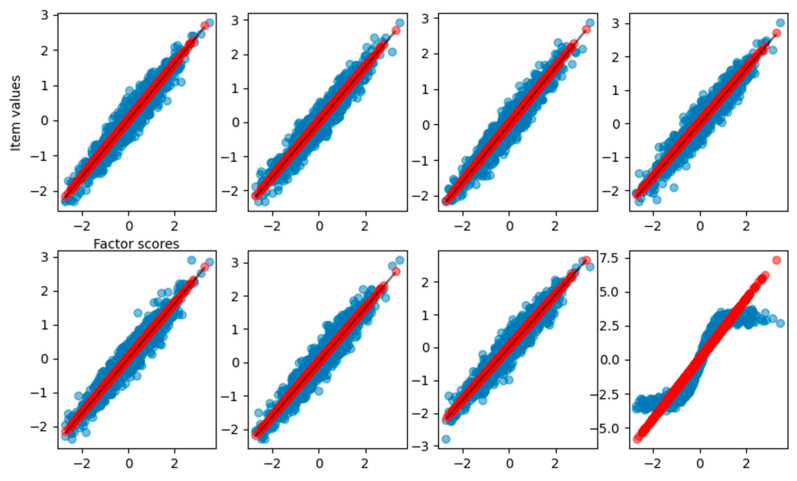
Graphical representation of the relationship between the factor scores and the eight-item values for the FA reconstruction with the dataset having one nonlinear item. Blue dots are the true item values, red points are the reconstruction from factor analysis, and the black solid line is the result of the curve fitting.

As we can see, the loadings of item number eight returned from FA are completely different from the right loading. In Figure 6, the graphical representation is reported.

**Figure 7:**
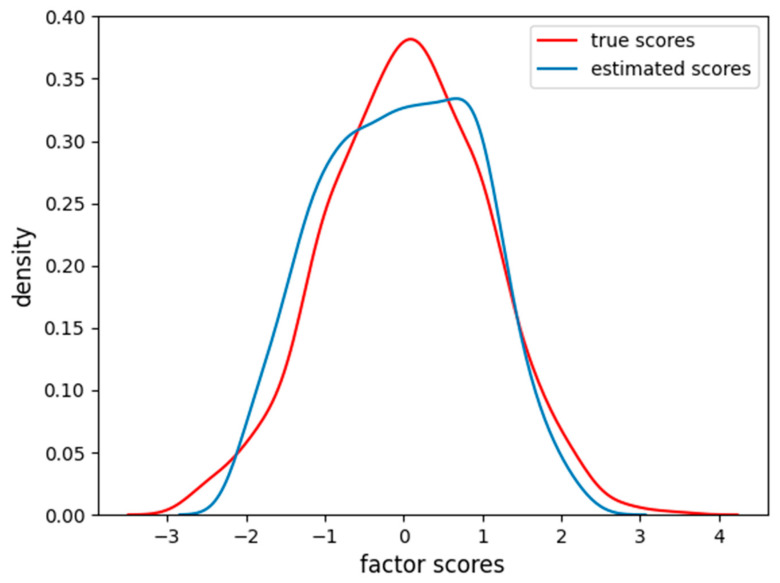
Density of true and estimated factor scores of the dataset with one item nonlinear using the FA. The curve follows a Gaussian distribution with mean of zero and unitary variance.

We can see that the last item–factor relationship, represented by the FA with a linear function, does not capture the correct relation. The fitting curve is not reported because it is not possible to perform the fit between a linear data reconstruction and a nonlinear sigmoidal curve. The explained variance is still pretty high, 0.88, but lower than before. In Figure 7 is reported the density of estimated factor scores and the true scores density. We can see that the estimation still resembles the true Gaussian distribution (MAE = 0.16, RMSE = 0.20) also if an item is wrongly reconstructed.

**Figure 8:**
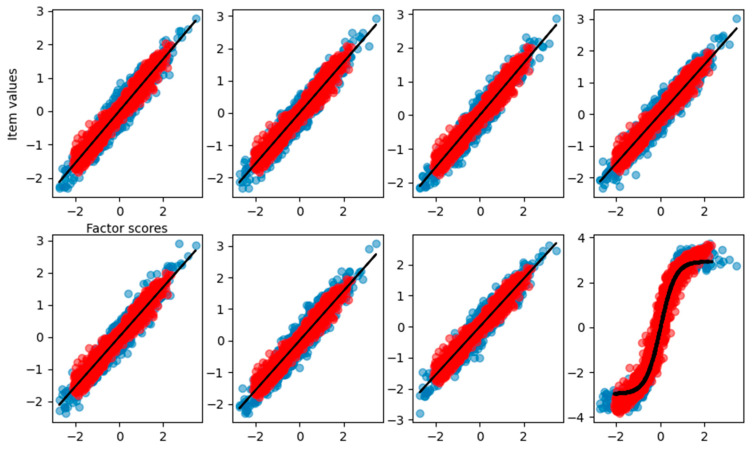
Graphical representation of the relationship between the factor scores and the eight-item values for the VAE using the dataset with one item nonlinear. Blue dots are the true item values, red points are the reconstruction from factor analysis, and the black solid line is the result of the curve fitting.

On the other hand, the VAE correctly captures the relationship between all the items and the factor, fitting all the items as well and retrieving the corresponding loadings, as used to generate the data (see Table 4 and Figure 8).

**Figure 9:**
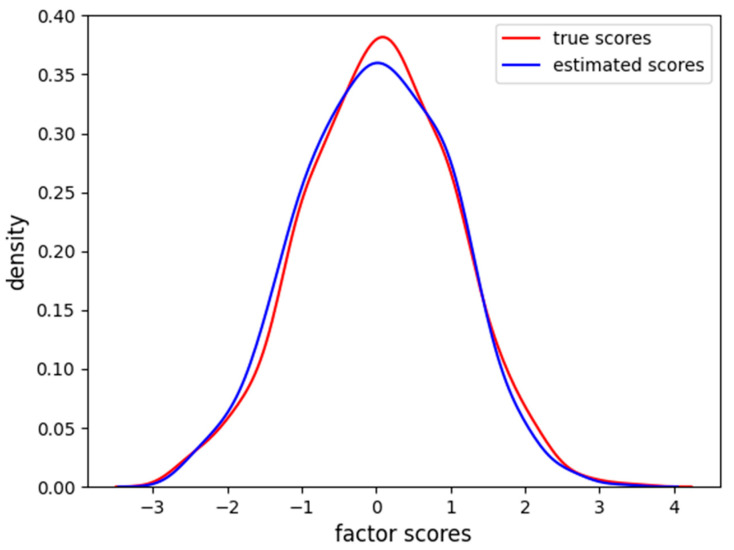
Density of true and estimated factor scores using the VAE with the dataset having one item nonlinear. The curve follows a Gaussian distribution with mean zero and unitary variance.

We can see how the last item is correctly approximated, and the fit is able to find the loading connecting the scores and the item values through the sigmoid function. The explained variance of the VAE is 0.92. In Figure 9, the density of the estimated factor scores is reported.

The Gaussian distribution is correctly identified with an MAE of 0.12 and RMSE of 0.16. The VAE is able to reconstruct the relationship and to estimate the factor scores also when the dataset is composed of one nonlinear item.

When the dataset is composed of a mixture of 50% of linear items and 50% of nonlinear items, we found that the FA correctly finds the loading for the linear items but completely fails on the nonlinear reconstruction of the remaining items (Table 5).

**Figure 10:**
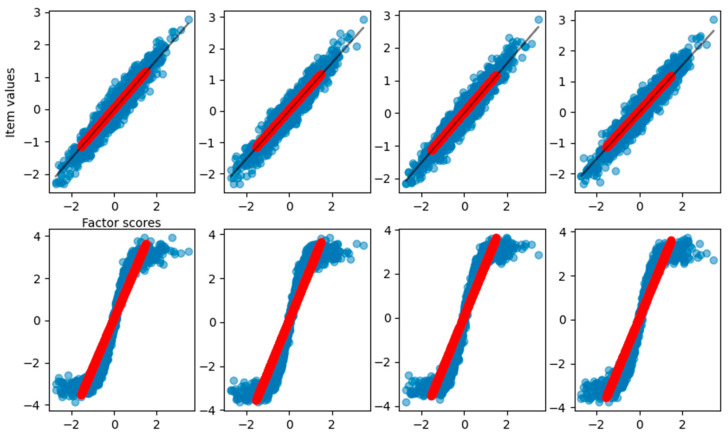
Graphical representation of the relationship between the factor scores and the eight-item values for the FA with the dataset having four items nonlinear. Blue dots are the true item values, red points are the reconstruction from factor analysis, and the black solid line is the result of the curve fitting.

From the graphical reconstruction showed in Figure 10, we can see how the FA tries to minimize the error, focusing mostly on the linear part of the sigmoid and leaving out item values related to high and low factor scores. We can see that also the linear items are reconstructed only in the central part of factor scores, leaving the more extreme values basically unpredictable.

**Figure 11:**
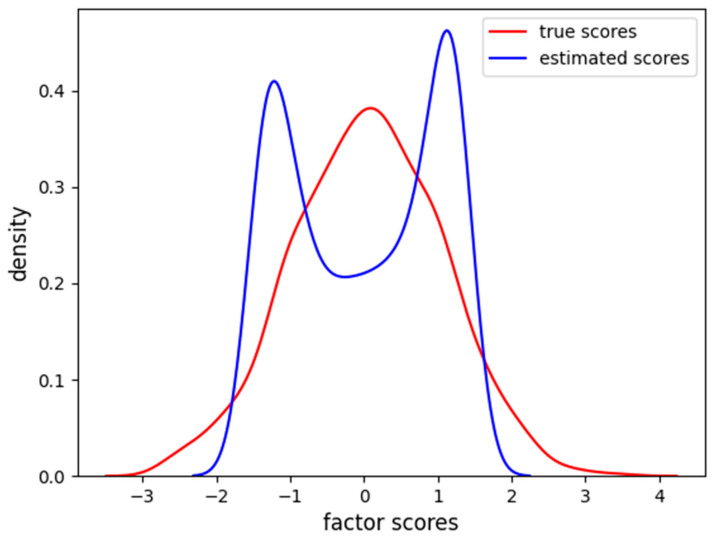
Density of true and estimated factor scores obtained using the FA using the dataset having four nonlinear items. The curve follows a Gaussian distribution with mean of zero and unitary variance.

The factor scores estimation is less accurate than before (MAE = 0.35, RMSE = 0.46), and, from Figure 11, we can see how there are two peaks in the density estimation, the two classes of the items (linear and sigmoidal) are probably separated into distinct latent factor scores and the FA is not able to find the correct Gaussian distribution of the factor scores.

**Figure 12:**
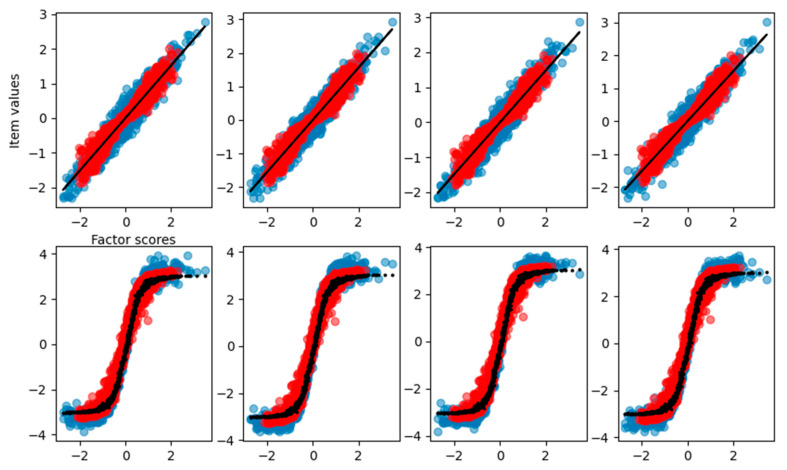
Graphical representation of the relationship between the factor scores and the eight-item values for the VAE with the dataset having four items nonlinear. Blue dots are the true item values, red points are the reconstruction from factor analysis, and the black solid line is the result of the curve fitting.

The VAE, on the other hand, correctly reconstructs all the item–factor relationships (Figure 12) and the right loadings (Table 6) with an explained variance of 0.92.

**Figure 13:**
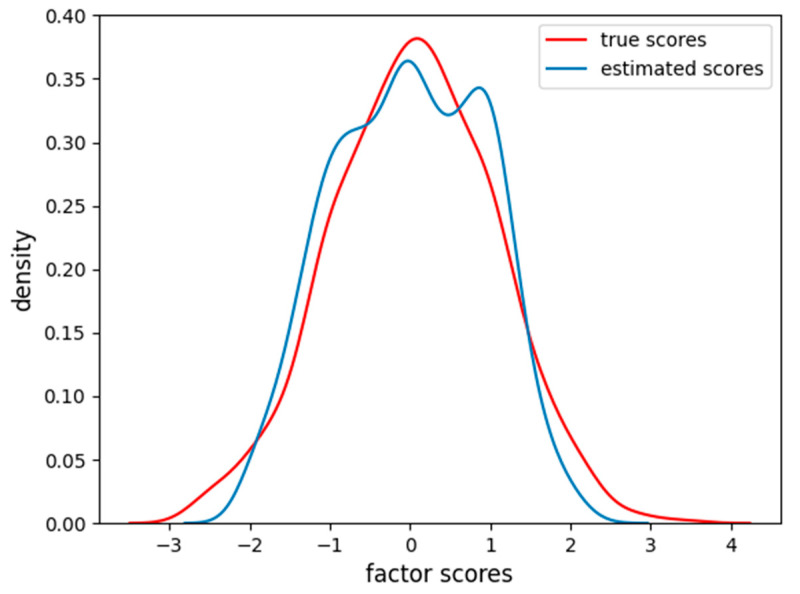
Density of true and estimated factor scores obtained using the VAE with the dataset having four nonlinear items. The curve follows a Gaussian distribution with mean of zero and unitary variance.

Furthermore, the VAE correctly infers the Gaussian distribution of the factor score, with an error just a little bit higher than in the previous, simpler experiments (MAE = 0.15, RMSE = 0.18) (Figure 13). The VAE is able to reconstruct the relation for a larger range of factor scores providing a more accurate reconstruction in both linear and nonlinear items.

### 4.3. Results Summary and Generalization to a Two-Factors Dataset

In this section, for clarity of reading, we present a table summarizing all previous results, including the explained variance of item reconstruction and the MAEs and RMSEs of factor scores retrieval in Table 7. Additionally, we include the results of experiments with a two-factor dataset in Table 8. The data generation procedure follows the same method as before: for each factor, eight related items are generated according to Equations (11) and (14) for linear and nonlinear relationships.

As shown in Table 8, the FA results are worse for two factors in terms of explained variance, while the VAE remains stable. This is due to the increased amount of nonlinearity in the dataset; in this case, each factor has one to four nonlinear items, doubling the total number of nonlinear items compared with the experiments presented in Table 1. The factor analysis performance in terms of factor score reconstruction is similar to the errors obtained with the one-factor reconstruction, likely because the number of nonlinear items related to each factor remains the same. We report the average MAE and RMSE between the two factors.

### 4.4. Discussion

In the study described in the previous paragraph, we tested a variational autoencoder on two datasets simulated from a factor-based population. The first dataset contained linear relationships between items and the factor, while the second dataset had nonlinear relationships.

Our results show that when the relationships between items and factors are linear, the VAE produces results comparable to linear factor analysis. Indeed, the VAE converged towards the factor analysis solution, accurately estimating the latent scores and giving the possibility to retrieve the factor loading values.

However, when the relationships between items and factors are nonlinear, the VAE outperforms factor analysis in terms of estimating latent scores and reconstructing the original data. In particular, the results show that the relationship between the internal nodes of the VAE and the reconstructed output approximates the function that defined the relationship between the items and the factors. By fitting this function, we obtained the values of the loadings, which are the parameters of the function.

One important limitation of such a study is that common psychometric data are often ordinal in nature. In the context of psychometric data analysis, several methods, such as polychoric correlation and weighted least-squares estimation techniques, have been proposed to deal with ordinal data in the linear case.

Moreover, while we simulated a sigmoid function in this study and successfully retrieved the factor loading values, the relationships between items and factors in real cases may exhibit more complex and peculiar forms. Consequently, research should focus on exploring how VAEs learn these intricate relationships.

So, it seems clear that testing the VAE on real datasets is dutiful to evaluate its performance under more realistic conditions and consequently to test its applicability in practical situations.

In the next study presented, we explore the application of VAEs on real and ordinal data, considering both linear and nonlinear relationships among items and factors.

## 5. Real Case Analysis

### 5.1. Emotional Stability in Teachers across Cultures

The real data used in the present study to compare the variational autoencoder with factor analysis come from a study by Vallone et al. [47] and consists of responses from 589 teachers to the items of the “Emotional Stability” scale, belonging to the short form of the Multicultural Personality Questionnaire (MPQ-SF; Van Der Zee, Van Oudenhoven, Ponterotto, and Fietzer [48]).

Regarding the participants’ characteristics, 29.4% (n = 173) were male, and 70.6% (n = 416) were female. Most of the teachers were over 45 years old (age over 45 years: n = 291, 49.4%; between 35 and 45 years: n = 144, 24.5%; under 35 years: n = 154, 26.1%) and highly experienced (working seniority over 10 years: n = 338, 57.4%; between 5 and 10 years: n = 121, 20.5%; under 5 years: n = 130, 22.1%). Regarding their country of origin, 18.5% of the participants (n = 109) were from Austria, 19.7% (n = 116) from Belgium, 7.1% (n = 42) from Germany, 18.5% (n = 109) from Italy, and finally, 36.2% (n = 213) were from Spain.

The short version of the Multicultural Personality Questionnaire assesses five key personality characteristics closely related to the general personality scales of the Big Five [49].

The “Emotional Stability” factor of the MPQ-SF is measured on a 5-point Likert scale (1 = not applicable at all, 5 = completely applicable) used to respond to 8 personal descriptors (e.g., “Keeps calm when things don’t go well”) attached to the following statement: “To what extent do the following statements apply to you?” A higher score represents a higher presence of the multicultural personality trait.

The MPQ-SF has been adopted and tested in various populations (e.g., teachers, students, employees, spouses/children of expats, immigrants) and cultures (e.g., Spain, Italy, Germany, Britain, Netherlands, United States, Canada, Singapore, Australia, New Zealand, China), including the countries involved in the study from which the data come from [50]. In all cases, the questionnaire has received wide empirical support internationally as one of the most valid and robust measures for assessing multicultural personality and, more generally, intercultural competence [49,50].

In the study of Vallone et al. [47], Cronbach’s α values for the MPQ-SF scales were all acceptable. The mean and the standard deviation of participants’ scores on Emotional Stability descriptors are 3.26 and 0.61, respectively.

#### 5.1.1. Real Case Results

In this section, we reconstruct the relationship between latent factors and the item response in a real-world scenario. Differently from the previous section, here we do not know the true relationships that link the item scores and the latent factor, and we use the VAE as a universal function approximator to reconstruct this relationship. To reconstruct this relationship, we used the VAE as a reconstruction model and compared the results with the reconstruction given from the factor analysis. Given that classical methods of analysis, like factor analysis, fail to capture the nonlinearity present in the data, we explore the item–factor relationship in search of nonlinearity, if any, with the VAE. The VAE hyperparameters are the same as discussed in the Methods section, and we only changed the number of hidden neurons, set to eight.

**Figure 14:**
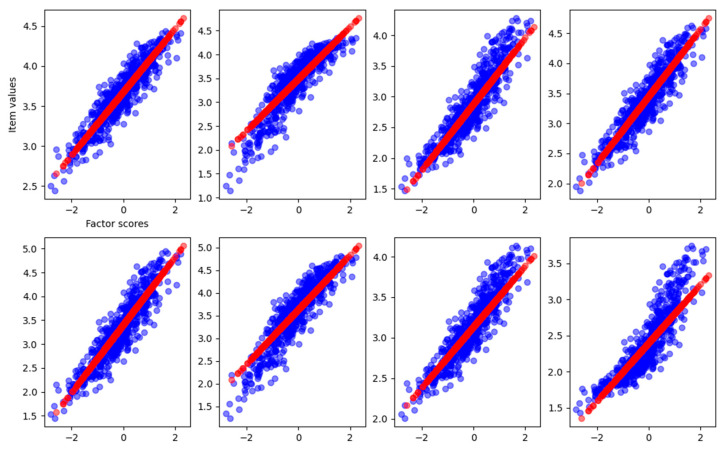
Graphical representation of the relationship between the factor scores and the eight-item values as reconstructed from the VAE and the FA. Blue dots are the VAE reconstruction, and red points are the reconstruction from factor analysis.

Figure 14 shows the reconstructed relation between the latent factor and the eight items from the VAE and the FA.

From Figure 14, it is clear how not all items are linearly connected with their underlying factor. We can clearly see how the VAE evidences a strong nonlinear relationship between the latent factor and several items. For clarity, the FA loadings for each item are reported in Table 9, along with the loadings that we retrieve fitting the VAE with a linear function.

We stress the fact that the factor scores in the case of the VAE are directly computed from the latent layer of the network, while in the case of FA, we use the maximum likelihood method to estimate them. The FA fails to correctly reconstruct the relations and tries to minimize the error approximating the nonlinearity of the true relation.

#### 5.1.2. Discussion

Our results on the real-world dataset confirm the potential of variational autoencoders (VAE) in reconstructing the intricate relationship between latent factors and item responses. It is worth noting that in contrast to the previous section, where we had the advantage of knowing the true relationship due to simulation, the true shape of the relationship remains unknown in this real-world scenario. However, having demonstrated the VAE’s ability to reconstruct the correct shape of the function linking items and factors in the previous section, we can reasonably hypothesize that the shape discovered by the autoencoder mirrors this relationship.

In this particular case, we observed that certain items exhibit a curvilinear relationship with the latent factor. Interestingly, some of these items show relatively low factor loadings.

In a traditional factor analytic approach, items with lower loadings are often labeled as less informative or weakly associated with latent factors. However, our findings suggest a different perspective. Some of the items with lower factor loadings may not be inherently less informative; rather, they might have unique and nonlinear relationships with latent factors that elude linear techniques.

This perspective carries substantial implications for psychometric research and data analysis in a broader context. It underscores the critical importance of incorporating nonlinear modeling approaches when investigating the connections between latent variables and observed indicators.

Integrating nonlinear modeling techniques like VAE can offer researchers the possibility to have a deeper and more informative insight into the complex relationships between latent factors and observed items, enriching their understanding of item characteristics.

## 6. General Conclusions

The research presented in this paper has aimed to explore the capabilities of variational autoencoders (VAEs) in the realm of psychometric data analysis. Our investigations sought to evaluate the performance of VAEs in capturing both linear and nonlinear relationships between items and latent factors. This involved testing the VAE’s ability to accurately estimate latent scores and reconstruct original data using both simulated and real psychometric data.

The findings from these studies suggest that VAEs could offer valuable contributions to the study of psychometric data dimensionality, complementing traditional psychometric data analysis by effectively capturing nonlinear relationships among items and factors. When dealing with linear relationships, these models produced results similar to those of linear factor analysis. However, the true advantages of VAEs emerged in the presence of nonlinear relationships. The VAE’s ability to reconstruct original data more accurately than conventional methods opens up possibilities for developing psychometric questionnaires that include items that do not conform to the assumptions of classical psychometric methods yet provide valuable insights and enhance our understanding of the constructs being measured.

In simulated cases, the VAE demonstrated an ability to estimate latent scores more accurately than linear factor analysis, which directly impacts the measurement of psychological constructs. This ability reduces the risk of obtaining false knowledge about the quantities of latent traits possessed by subjects and of incorrectly identifying the behaviors to expect from individuals based on their latent traits, thus providing a more comprehensive and nuanced understanding of individuals.

Future research should continue to explore and refine the use of VAEs in psychometric analysis, addressing the challenges related to model interpretability and accuracy.

## Figures and Tables

**Table 1:** Fitting loadings and FA loadings for the linear case scenario are reported for each item, along with their errors. Communalities from the FA are also reported.

	Item 1	Item 2	Item 3	Item 4	Item 5	Item 6	Item 7	Item 8
Fitting loadings	0.80 ± 0.01	0.80 ± 0.01	0.80 ± 0.01	0.80 ± 0.01	0.80 ± 0.01	0.80 ± 0.01	0.80 ± 0.01	0.80 ± 0.01
FA loadings	0.80 ± 0.02	0.80 ± 0.02	0.80 ± 0.02	0.80 ± 0.02	0.80 ± 0.02	0.80 ± 0.02	0.80 ± 0.02	0.80 ± 0.02
Communalities	0.95 ± 0.03	0.95 ± 0.03	0.95 ± 0.04	0.95 ± 0.03	0.95 ± 0.03	0.95 ± 0.03	0.95 ± 0.03	0.95 ± 0.04

**Table 2:** Fitting loadings for each item using the VAE in the linear case scenario, fitting standard deviation is also reported.

	Item 1	Item 2	Item 3	Item 4	Item 5	Item 6	Item 7	Item 8
Loadings	0.80 ± 0.01	0.81 ± 0.01	0.79 ± 0.01	0.80 ± 0.01	0.80 ± 0.01	0.79 ± 0.01	0.80 ± 0.01	0.80 ± 0.01

**Table 3:** Fitting loadings and FA loadings obtained with the FA with the dataset having one nonlinear item, results are reported for each item along with their errors. Communalities from the FA are also reported. For items identified with the asterisk, fitting is not performed due to the impossibility of fitting linear data according to the true sigmoidal generative function.

	Item 1	Item 2	Item 3	Item 4	Item 5	Item 6	Item 7	Item 8
Fitting loadings	0.79 ± 0.02	0.81 ± 0.02	0.81 ± 0.02	0.79 ± 0.02	0.81 ± 0.02	0.79 ± 0.02	0.80 ± 0.01	*
FA loadings	0.81 ± 0.03	0.80 ± 0.03	0.80 ± 0.03	0.81 ± 0.03	0.81 ± 0.03	0.81 ± 0.03	0.80 ± 0.02	2.35 ± 0.05
Communalities	0.95 ± 0.03	0.95 ± 0.03	0.95± 0.04	0.95 ± 0.03	0.95 ± 0.03	0.95 ± 0.03	0.95 ± 0.03	0.47 ± 0.04

**Table 4:** Fitting loadings for each item obtained using the VAE with a dataset having one item nonlinear, fitting standard deviation is also reported.

	Item 1	Item 2	Item 3	Item 4	Item 5	Item 6	Item 7	Item 8
Loadings	0.81 ± 0.01	0.79 ± 0.01	0.80 ± 0.01	0.81 ± 0.01	0.79 ± 0.01	0.79 ± 0.01	0.80 ± 0.01	0.81 ± 0.01

**Table 5:** Fitting loadings and FA loadings obtained with the FA using the dataset having four nonlinear items, results are reported for each item along with their errors. Communalities from the FA are also reported. For items identified with the asterisk, fitting is not performed due to the impossibility of fitting linear data according to the true sigmoidal generative function.

	Item 1	Item 2	Item 3	Item 4	Item 5	Item 6	Item 7	Item 8
Fitting loadings	0.74 ± 0.02	0.75 ± 0.02	0.75 ± 0.02	0.75 ± 0.02	*	*	*	*
FA loadings	0.81 ± 0.03	0.80 ± 0.03	0.80 ± 0.03	0.81 ± 0.03	2.32 ± 0.05	2.28 ± 0.05	2.47 ± 0.05	2.24 ± 0.05
Communalities	0.95 ± 0.03	0.95 ± 0.03	0.95 ± 0.04	0.95 ± 0.03	0.32 ± 0.07	0.35 ± 0.06	0.33 ± 0.05	0.34 ± 0.06

**Table 6:** Fitting loadings for each item obtained using the VAE with the dataset having four nonlinear items, fitting standard deviation is also reported.

	Item 1	Item 2	Item 3	Item 4	Item 5	Item 6	Item 7	Item 8
Loadings	0.78 ± 0.01	0.79 ± 0.01	0.82 ± 0.01	0.81 ± 0.01	0.79 ± 0.01	0.79 ± 0.01	0.81 ± 0.01	0.81 ± 0.01

**Table 7:** MAE and RMSE for the factor score reconstruction and explained variance for the item reconstruction of the two methods. Different levels of nonlinearity in the dataset are reported.

	VAE Linear	FA Linear	VAE One Item Nonlinear	FA One Item Nonlinear	VAE Four Item Nonlinear	FA Four Item Nonlinear
MAE and RMSE factor scores reconstruction	0.13, 0.17	0.11, 0.15	0.12, 0.16	0.16, 0.20	0.15, 0.18	0.35, 0.46
Explained variance item reconstruction	0.91	0.92	0.92	0.88	0.92	0.80

**Table 8:** MAE and RMSE for the factor scores reconstruction and Explained variance for the item reconstruction of the two methods with a two-factors dataset. Different levels of nonlinearity in the dataset are reported.

	VAE Linear	FA Linear	VAE One Item Nonlinear	FA One Item Nonlinear	VAE Four Item Nonlinear	FA Four Item Nonlinear
MAE and RMSE factor scores reconstruction	0.12, 0.16	0.10, 0.15	0.11, 0.17	0.17, 0.21	0.14, 0.18	0.37, 0.49
Explained variance item reconstruction	0.91	0.92	0.92	0.85	0.91	0.66

**Table 9:** Loadings found by factor analysis on the real dataset, results are reported along with the error.

	Item 1	Item 2	Item 3	Item 4	Item 5	Item 6	Item 7	Item 8
FA loadings	0.48 ± 0.04	0.52 ± 0.03	0.56 ± 0.03	0.59 ± 0.03	0.69± 0.03	0.60 ± 0.03	0.40 ± 0.04	0.41 ± 0.04
VAE loadings	0.52 ± 0.05	0.62 ± 0.04	0.53 ± 0.02	0.57 ± 0.04	0.67± 0.05	0.65 ± 0.03	0.40 ± 0.04	0.52 ± 0.04

## Data Availability

The data presented in this study are available on request from the corresponding author.
